# DKA management and outcomes

**DOI:** 10.1186/1687-9856-2013-S1-O16

**Published:** 2013-10-03

**Authors:** Francesco Chiarelli, M Loredana Marcovecchio

**Affiliations:** 1 Department of Paediatrics, University of Chieti, Chieti, Italy

## 

Diabetic ketocidosis (DKA) is an acute life threatening complication of type 1 diabetes mellitus (T1D). DKA is characterized by the the triad of hyperglycemia, metabolic acidosis and increased total body ketone concentration. These metabolic derangements result from the combination of absolute or relative insulin deficiency and increased levels of counter-regulatory hormones.

DKA is associated with both short-term risks and long-term consequences. Recent data have shown that DKA is responsible for up to 73% of causes of deaths during the first decade of diabetes. Mortality is predominantly related to the occurrence of cerebral edema, which occurs in 0.3-1% of patients, whereas only a minority of deaths in DKA is due to other causes.

DKA represents the initial manifestation of T1D in 13-80% of cases and it can also occur in 25% of cases of type 2 diabetes at onset. In addition, DKA is a common complication in patients with known diabetes, where it may be the consequence of illness, poor compliance, or malfunction of diabetes care equipment.

Early identification and treatment of DKA are two key points to minimizing the risks of this complication. Children with DKA should be treated in experienced centers and adherence to guidelines for the management of this condition is of paramount importance.

Treatment of DKA requires strict monitoring of the patient, correction of hyperglycemia, acidosis and ketosis and replacement of fluid and electrolytes losses.

Another important point is the identification and treatment of precipitating events.

Early recognition of signs indicative of cerebral edema is essential to prevent the morbidity and mortality associated with this complication. Cerebral edema occurs in 0.3-1% of patients in DKA and represents the most common cause of mortality in children with DKA, accounting for 60-90% of all DKA deaths. In addition, 10-25% of survivors have significant residual morbidity. The etiology of cerebral edema is poorly understood, but it is likely related to vasogenic, osmotic, and ischemic mechanisms.

Prevention of DKA at diagnosis is of paramount importance and should be based on intensive community interventions and education of health care providers to raise awareness. In addition, preventive strategies should be applied to avoid episodes of DKA in patients with an already known diagnosis of diabetes. This requires patient education and access to specific diabetes programs and services.
